# Comparative genomics and experimental evolution of *Escherichia coli* BL21(DE3) strains reveal the landscape of toxicity escape from membrane protein overproduction

**DOI:** 10.1038/srep16076

**Published:** 2015-11-04

**Authors:** Soon-Kyeong Kwon, Seong Keun Kim, Dae-Hee Lee, Jihyun F. Kim

**Affiliations:** 1 Department of Systems Biology and Division of Life Sciences, Yonsei University, 50 Yonsei-ro, Seodaemun-gu, Seoul 120-749, Republic of Korea; 2 Synthetic Biology and Bioengineering Research Center, Korea Research Institute of Bioscience and Biotechnology (KRIBB), 125 Gwahak-ro, Yuseong-gu, Daejeon 305-806, Republic of Korea; 3 Biosystems and Bioengineering Program, University of Science and Technology (UST), 217 Gajung-ro, Yuseong-gu, Daejeon 305-350, Republic of Korea

## Abstract

Achieving sufficient yields of proteins in their functional form represents the first bottleneck in contemporary bioscience and biotechnology. To accomplish successful overexpression of membrane proteins in a workhorse organism such as *E. coli*, defined and rational optimization strategies based on an understanding of the genetic background of the toxicity-escape mechanism are desirable. To this end, we sequenced the genomes of *E. coli* C41(DE3) and its derivative C43(DE3), which were developed for membrane protein production. Comparative analysis of their genomes with those of their ancestral strain *E. coli* BL21(DE3) revealed various genetic changes in both strains. A series of *E. coli* variants that are able to tolerate transformation with or overexpression of membrane proteins were generated by *in vitro* evolution. Targeted sequencing of the evolved strains revealed the mutational hotspots among the acquired genetic changes. By these combinatorial approaches, we found non-synonymous changes in the *lac* repressor gene of the *lac* operon as well as nucleotide substitutions in the *lac*UV5 promoter of the DE3 region, by which the toxic effect to the host caused by overexpression of membrane proteins could be relieved. A mutation in *lacI* was demonstrated to be crucial for conferring tolerance to membrane protein overexpression.

Membrane proteins comprise a significant percentage of total cellular proteins and are responsible for a variety of essential cellular processes, including nutrient and waste transport across the membrane, signal transduction, cell-to-cell communication, and maintenance of cell morphology^1^. Thus, it is not surprising that malfunctions of integral membrane proteins are implicated in numerous human diseases and that they represent targets for many pharmaceuticals^2^. Despite their importance, the tremendous difficulties involved in their structural analysis and biochemical and functional characterization have resulted in their underrepresentation in structural databases. These difficulties are compounded by the fact that the abundance of membrane proteins is often too low for them to be isolated in sufficient amounts from a native host system. Therefore, heterologous expression is the primary route used to produce sufficient amounts of membrane proteins for biochemical, biophysical, and structural studies. However, the production of recombinant membrane proteins in heterologous hosts, especially *E. coli*, often results in growth retardation or even host cell death due to the highly hydrophobic sequences required for their insertion into the membrane^3^.

Various approaches have been taken in the attempt to successfully produce membrane proteins in *E. coli*, including transposon mutagenesis^4^, chemical/*mutD5* mutagenesis^5^, ASKA library overexpression^6^,^7^, membrane protein biogenesis pathway engineering^4^, chaperone pathway engineering^8^, glucose uptake suppression^9^, and modulating the activity of the T7 RNA polymerase (RNAP) by the T7 lysozyme^10^,^11^. However, rational strategies for engineering the host system are often very difficult due to the complex process of membrane protein biogenesis (including synthesis, folding, and insertion), which needs to be coordinated and balanced. However, a selection-based method that uses an “*in vitro* evolution” approach may bypass the complexity of the biogenesis process and its impact on the host^12^,^13^,^14^.

Miroux and Walker employed this selection-based approach to reduce toxicity and improve the expression characteristics of membrane proteins using *E. coli* BL21(DE3), which is the most widely used overexpression vehicle^12^. The protein overexpression ability of BL21(DE3) depends on RNAP, which elongates the transcript eight times faster than *E. coli* RNA polymerase^15^,^16^. The use of T7 RNAP enables higher expression of a T7 promoter-governed recombinant protein. The T7 RNAP gene carried by the DE3 prophage located in the chromosome, is controlled by the *lac*UV5 promoter, a variant that is stronger than the wild-type *lac* promoter^17^. In a selection-based approach, BL21(DE3) cells expressing a particular membrane protein were spread on solid medium in the presence of an inducer, and the surviving cells that could cope with the toxic effects of membrane protein overexpression were then selected. *E. coli* C41(DE3) is a derivative of BL21(DE3) that showed improved production of bovine oxoglutarate-malate transport protein (OGCP). *E. coli* C43(DE3) was found among C41(DE3) isolates that were tolerant to the overexpression of *E. coli* F-type ATPase subunit b (Ecb). C41(DE3) and C43(DE3), commonly known as the Walker strains, are currently widely used for production of a variety of membrane proteins and toxic proteins^18^. Subsequently, Wagner and colleagues observed that the Walker strains suffered less from Sec translocon saturation, which causes the aggregation of endogenous proteins in the cytoplasm and results in perturbation of the membrane proteome^19^. They also reported that three nucleotide changes in the –10 and +1 regions of the *lac*UV5 promoter in the Walker strains are responsible for slowing transcription by T7 RNAP, thus improving membrane protein overexpression by reducing host toxicity^11^. Based on these findings, they developed the Lemo(DE3) system, which allows T7 RNAP activity to be tuned using the T7 lysozyme, its natural inhibitor^11^,^20^. Alfasi and colleagues also demonstrated that mutations at the regulatory region of the T7 RNAP gene accumulate in BL21(DE3) during production of recombinant proteins^21^.

Although the mutations in the *lac*UV5 promoter in the Walker strains emerged as a rescue factor for cell viability, the mechanism(s) involved in reducing the toxicity are not fully understood. In addition to the mutations in the *lac*UV5 promoter or in the T7 RNAP gene, altered regulation by the *lac* repressor LacI or its operator site may affect T7 RNAP activity. Moreover, completely unexpected factor(s) may govern the membrane protein-induced kinetics of the Walker strains^10^. In this study, we employed comparative genomics and experimental evolution approaches to identify mechanisms associated with escape from the toxicity caused by membrane protein overexpression. We identified all of the genetic changes in the Walker strains through comparative genome-wide sequence analysis with BL21(DE3). We then defined two mutation hotspots among the toxicity-escaping variants obtained by *in vitro* evolution and revealed two classes of mutations responsible for the stable membrane protein-producing phenotype. A mutation in *lacI* was introduced into the parental strain to confirm that it was crucial for tolerance to membrane protein overexpression. Finally, we discuss the possible molecular mechanisms that explain the role of the LacI mutation in gene expression and toxicity escape.

## Results and discussion

### Comparative genomic analysis of *E. coli* strains BL21(DE3), C41(DE3), and C43(DE3)

**Figure 1:**
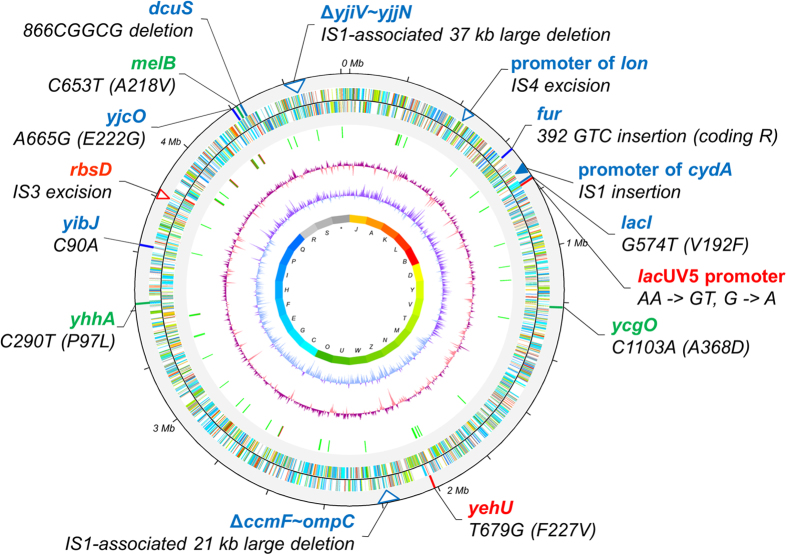
Genomic variations in *E. coli* C41(DE3) and C43(DE3) as compared to the BL21(DE3) backbone sequence in a circular representation. Mutations found in both C41(DE3) and C43(DE3) are depicted in red, C41(DE3) in green, and C43(DE3) in blue, respectively. Small-scale changes like SNPs or small DIPs are indicated with solid lines, and IS element-mediated large scale insertions or deletions with triangles.

To search for genetic factors that might be involved in the expanded capacity of strains C41(DE3) and C43(DE3) for the overexpression of membrane proteins, we sequenced the genomes of these two BL21(DE3) derivatives and examined genetic changes such as single nucleotide polymorphisms (SNPs), deletions, insertions or other polymorphisms (DIPs). We found seven and twelve genomic loci in C41(DE3) and C43(DE3), respectively, that were changed with respect to the ancestral BL21(DE3) strain (Table 1 and Fig. 1). Three SNPs in the *lac*UV5 promoter region of the T7 RNAP gene and one SNP each in the coding sequences of the *yehU* and *rbsD* genes were common to both C41(DE3) and C43(DE3). The mutations in *lac*UV5 were identical to those previously reported by Wagner and coworkers^11^. The *yehU* gene encodes the sensor kinase of a two-component system whose cognate response regulator is YehT^22^. The *rbs* operon, which is involved in high-affinity D-ribose transport^23^, is non-functional in BL21(DE3) due to an insertion of the IS*3* element in the first gene of the operon (*rbsD*)^24^. However, the IS*3* element was precisely excised from *rbsD* in C41(DE3) and C43(DE3).

The three non-synonymous mutations present only in C41(DE3) were located in the coding regions of *melB, ycgO*, and *yhhA*. These mutations may have appeared in C41(DE3) but were then lost when C43(DE3) evolved, or they may have arisen during subculturing of C41(DE3); thus, we speculate that they may not play important roles in the overexpression of membrane proteins in C41(DE3). More diverse genomic variations were detected in the C43(DE3) genome (Table 1 and Fig. 1). Among the C43(DE3)-specific genomic changes, SNPs were observed in the *lacI*, *yibJ*, and *yjcO* genes. The mutations in *lacI* and *yjcO* were non-synonymous, whereas the mutation in *yibJ* was synonymous. There are two *lacI* genes in the chromosomes of BL21(DE3) and its two derivatives, C41(DE3) and C43(DE3). The first is located adjacent to the *lac* operon, while the second is part of DE3 (a defective prophage inserted into the λ *att* site of *E. coli* BL21)^16^. The *lacI* mutation in C43(DE3) occurred in the DE3 region. The function of the *yjcO* gene has not been assigned. In addition to SNPs, several DIPs were observed in C43(DE3). The tri-nucleotide GTC was duplicated in the *fur* gene, while one of the two CGCCG sequences duplicated in BL21(DE3) in the *dcuS* gene was deleted, thus shifting the gene back in frame. Interestingly, according to Schlegel and coworkers, *dcuS* reversion is reported to be present also in C41(DE3), suggesting that this mutation might be easily selected in response to stress conditions^10^. Moreover, an IS*1* element was inserted into the promoter region of *cydA*, and an IS*4* element was excised from the promoter region of *lon*. Two large-scale genomic deletions in C43(DE3), across *ccmF* ~ *ompC* and *yjiV* ~ *yjjN*, seemed to be associated with IS*1*. The *yjiV* ~ *yjjN* region is related to foreign DNA restriction. The *mcrC* ~ *mrr* genes in this region are deleted in some commercial *E. coli* strains such as DH10B or TOP10 and also eliminated in C43(DE3) by a large 35-kb deletion event.

To distinguish genuine mutations from clonal variations among the detected SNPs and DIPs, we randomly selected ten colonies of C41(DE3) and C43(DE3) and conducted targeted sequencing of the corresponding regions with the exception of the two large genomic deletions. No clonal variability was observed among these regions.

### Identification of mutations responsible for the overexpression of membrane proteins in C41(DE3) and C43(DE3)

Mutations in the *rbs* operon, the *yehU* structural gene, and the *lac*UV5 promoter of the T7 RNAP gene were common to C41(DE3) and C43(DE3). Recently, Schlegel *et al.* showed that the superior capacity of C41(DE3) for OGCP overexpression was due to mutations in the *lac*UV5 promoter^10^. We also doubted that the mutations in *rbsD* and *yehU* were also responsible for the toxicity-escape phenotype. To test their roles in toxicity escape, the *rbsD* and *yehU* sequences in BL21(DE3) were individually replaced with those from C41(DE3) and C43(DE3) by homologous recombination mediated by the λ Red system (See Methods). The BL21(DE3) mutants, when transformed with pMW7(OGCP), could not endure the overexpression of OGCP (data not shown), indicating that these mutations are not directly related to escaping toxicity during membrane protein overexpression. No role of *rbsD* in improved production of OGCP is consistent with the previous report^10^. Therefore, we concluded that the mutations in the *lac*UV5 promoter were exclusively responsible for tolerance to OGCP protein overexpression in C41(DE3). Weakened T7 RNAP activity due to mutations in the *lac*UV5 promoter may have saved the cells from the toxicity caused by uncoupling the transcription and translation of the target membrane proteins^11^,^12^.

Because there is no difference in the *lac*UV5 promoter region sequence between C41(DE3) and C43(DE3), mutations in the *lac*UV5 promoter cannot fully explain why C43(DE3) but not C41(DE3) could overcome the toxicity induced by the overexpression of membrane proteins such as Ecb. Therefore, we further investigated the C43(DE3)-specific mutations. Mutations in the structural genes *dcuS, fur, lacI,* and *yjcO* and in the promoters of *cydA* and *lon* in C43(DE3) were introduced into BL21(DE3) and C41(DE3) by homologous recombination. Overexpression of OGCP in BL21(DE3) and Ecb in C41(DE3) containing the individual mutations resulted in cell death in all strains including *lon*, except those possessing the mutation in *lacI*. Details on the effect of *lacI* mutations on membrane protein overexpression will be described and discussed in later sections.

Lon protease deficiency due to IS*4* insertion in the promoter region of *lon* is a well-known genetic feature of *E. coli* B^25^. However, *lon* can be induced in response to a stress such as heat shock^26^. The promoter activity of *lon* in C43(DE3) has apparently been restored due to IS*4* excision, as indicated by the detection of its transcript in an RT-PCR experiment (data not shown). This is in agreement with a previous observation that Lon protease is present in C43(DE3)^11^. The Lon protease plays an important role in the quality control of membrane-protein synthesis^27^. It rescues cells by clearing away misfolded or partially translated proteins that are stalled at the ribosome during the co-translational membrane-insertion process. Deactivation of the toxic system also allows cells to better recover from Sec pathway jamming. Although no obvious improvement in the expression of the monotopic Ecb protein was observed in a *lon* revertant of C41(DE3) in our hands, it remains a tempting candidate for rescuing cells from the overexpression of polytopic membrane proteins^28^,^29^. The restoration of Lon protease expression was reported to improve the growth of hosts overexpressing a polytopic membrane protein^30^.

### Frequently occurring mutations are located upstream of the T7 RNA polymerase gene in strains that overcome the transformation toxicity

The question remained whether the mutations in C41(DE3) and C43(DE3) emerged by chance or whether strains tended to acquire specific mutations endowing them with the ability to escape the toxic effect of membrane protein overexpression. To answer this question, a number of BL21(DE3) variants were obtained through parallel evolution in different batches. The repeated appearance of certain mutations in multiple batches would increase the likelihood that the corresponding genes are under selection pressure to circumvent toxicity.

Ecb and Ecb fused to green fluorescent protein (GFP) for convenient monitoring of protein expression levels, were used for the analysis. The fluorescence intensity of GFP correlated with the expression level of correctly folded membrane proteins^31^. To screen for evolved strains capable of overcoming the toxicity associated with transformation of Ecb, BL21(DE3) was transformed with pMW7(Ecb)^12^, or pMW7(Ecb-GFP), which express Ecb or GFP-fused Ecb, respectively, under the control of the T7 promoter. Two sub-populations, one comprising large colonies and the other comprising small colonies, appeared on L agar medium without IPTG. We chose fifteen large colonies that showed the transformation toxicity-escape phenotype for further studies.

**Figure 2:**
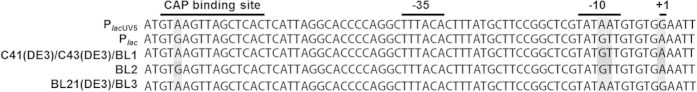
Differences in the promoter sequences that control the transcription of the T7 RNA polymerase gene between *E. coli* BL21(DE3) and fifteen mutant derivatives that overcame the transformation toxicity induced by a membrane protein-encoding gene. Three strains in the mutant group BL1 had the same promoter sequence as C41(DE3) and C43(DE3). Nine BL2 mutants had reverted the *lac*UV5 promoter sequence to that of the wild-type *lac* promoter. Three BL3 strains showed no difference from the *lac*UV5 promoter of BL21(DE3).

We analyzed the sequences of the region upstream of the T7 RNAP gene in the fifteen colonies, as mutations in the *lac*UV5 promoter are known to confer tolerance to the toxicity of membrane protein expression. Among the fifteen mutants, twelve harbored mutations upstream of the T7 RNAP gene, as expected. Interestingly, three of these mutants (Group BL1 in Table 2 and Fig. 2; termed BL for large colonies obtained from BL21(DE3)) had the same promoter sequences as C41(DE3) and C43(DE3), whereas the other nine mutants (Group BL2 in Table 2 and Fig. 2) had additional SNPs at a single position. This nucleotide change was located in the binding site of the catabolite activator protein (CAP), and it restored the *lac*UV5 promoter to the wild-type *lac* promoter sequence. The remaining three mutants (Group BL3 in Table 2 and Fig. 2) showed no sequence differences from BL21(DE3) in the promoter of the T7 RNAP. Because previous reports demonstrated that mutations in the gene encoding T7 RNAP make the enzyme less active^15^, we examined the protein-coding sequence of T7 RNAP. Using targeted sequencing of the T7 RNAP gene in the three BL groups, we identified a nucleotide deletion at C660 that caused a frame shift, a C805T substitution that led to an early termination of translation, and a C2371T substitution that changed the amino acid sequence to L791F in the three BL3 mutants.

In addition to the three nucleotide changes at the −10 and +1 regions of the *lac*UV5 promoter, the identification of additional genetic changes in the CAP binding site in the region upstream of T7 RNAP and in the T7 RNAP structural gene in the BL strains indicate that these regions are mutation hotspots. In response to the toxicity induced by membrane proteins, the BL1 and BL2 strains have evolved to carry mutations in the *lac*UV5 region that are similar to those observed in C41(DE3) or C43(DE3). The complete conversion of the *lac*UV5 promoter to the wild-type *lac* promoter upstream of the T7 RNAP gene in BL2 should contribute to higher basal-level production of Ecb compared with BL1, BL3, C41(DE3) or C43(DE3) under uninduced conditions (data not shown). Our results also demonstrate that mutations in the T7 RNAP gene *l* of BL3 may overcome the cellular toxicity caused by leaky expression of the membrane protein. By inducing the T7 RNAP mutation, the host can modulate the expression of a toxic protein to various degrees. Indeed, T7 RNAP truncation caused by frame shifting or by the introduction of a premature stop codon has been shown to decrease toxic protein expression^32^.

### Mechanism of the mutations upstream of the T7 RNA polymerase gene

The frequency of mutation in the *lac*UV5 promoter region of the T7 RNAP gene (87%, 12 out of 15) was unexpectedly high, considering that three to four nucleotides had to undergo concurrent mutation. Because we had previously sequenced and analyzed the genome of *E. coli* BL21(DE3)^17^, we again probed the genome sequence of BL21(DE3) to investigate this highly frequent mutation. Indeed, the promoter region of the T7 RNAP gene (P_*lac*UV5_ in Fig. 2) had nearly the same 1,723-bp DNA sequence as that of the *lac* operon (P_*lac*_ in Fig. 2), with the exception of 4 nucleotides. Therefore, it is possible that the mutation in the *lac*UV5 promoter arose from homologous recombination between the promoter regions of the T7 RNAP gene and the *lac* operon of BL21(DE3).

To test this possibility, we constructed BL21(DE3)Δ*lacI-*P_*lac*_ and C41(DE3)Δ*lacI-*P_*lac*_, in which *lacI*, the *lac* promoter, and part of *lacZ* were deleted to prevent recombination between the homologous sequences. We then repeated the evolution experiment by transforming the bacteria with pMW7(Ecb-GFP). In contrast to C41(DE3)Δ*lacI-*P_*lac*_, BL21(DE3)Δ*lacI-*P_*lac*_ barely survived the transformation (Supplementary Fig. 1). Only four colonies from BL21(DE3)Δ*lacI-*P_*lac*_ harboring pMW7(Ecb-GFP) arose and exhibited Ecb expression (BR strains in Supplementary Table S3; termed BR for BL21(DE3) recombinants). None of these strains contained mutations in the *lac*UV5 promoter region including the CAP binding site, but all contained non-synonymous mutations in the T7 RNAP structural gene. The last mutation was identified in two of the evolved strains. Thus, we concluded that the high mutational frequency of the *lac*UV5 promoter in BL21(DE3) cells overexpressing toxic membrane proteins is mediated by homologous recombination. Schlegel and colleagues also demonstrated that RecA-mediated recombination between P_*lac*UV5_ and P_*lac*_ drives the mutations in *lac*UV5 promoter in GFP overproducing BL21(DE3). In their study, no mutations in P_*lac*UV5_ were observed in the P_*lac*_ or *recA* knock-out strains^10^.

### Correlation between mutations in the *lac* repressor gene and protein expression capacity

The experimental evolution experiment described in the previous section was restricted to screening for mutants that could overcome the toxicity caused by transformation with pMW7(Ecb). We also performed an additional evolution experiment to obtain spontaneous mutants that overcame the toxicity caused by the induction of Ecb expression after IPTG treatment.

C41(DE3) was used as the parental strain for the evolution experiment, as wild-type BL21(DE3) cannot endure even basal-level expression of Ecb after transformation. Furthermore, the use of C41(DE3) avoided the frequent reversion events of the *lac*UV5 promoter to the *lac* promoter that occur when BL21(DE3) is used. The plasmid pMW7(Ecb-GFP) was transformed into C41(DE3), and the transformants were cultivated on L agar plates supplemented with ampicillin (Amp) in the presence of 0.1 mM IPTG. Among the resulting colonies, three showed growth and fluorescence intensity similar to C43(DE3) during subsequent cultivation. We named the mutants CL (large colonies obtained from C41(DE3)).

Because comparative genomic analysis and site-directed mutagenesis indicated that a C43(DE3)-specific mutation in the *lacI* gene enabled C41(DE3) to overexpress Ecb, we focused on mutations in *lacI* among the CL strains (Table 1). BL21(DE3) and its derivatives contain two copies of *lacI* in their genomes. One is located in the *lac* operon, while the second is present in the DE3 region. Sequencing both copies of *lacI* in the three evolved strains revealed that all possessed non-synonymous mutations in *lacI* (Table 3). Interestingly, CL1 and CL2 contained mutations in the *lacI* gene in the DE3 region and in the *lac* operon, respectively, while CL3 had *lacI* mutations in both regions. Several evolved CL strains with mutations in their *lacI* genes successfully overexpressed the Ecb protein. In the case of C43(DE3), the onset of protein production was delayed as compared to C41(DE3)^12^. Less protein was produced per cell in C43(DE3) than in C41(DE3); however, the total amount of protein produced per culture was higher in C43(DE3) because the cells continued to divide after the induction of gene expression. In conclusion, the core mutations that arose during the evolution from C41(DE3) to C43(DE3) and in the CL strains were located in the *lacI* gene.

### A mutation in the *lac* repressor gene of C41(DE3) restores toxic membrane protein expression capacity to a level comparable with C43(DE3)

Now widely used pET-vectors harbor *lacI* and *lacO* in the T7 promoter region^15^,^16^,^33^, while pMW7 does not. Using a modified pMW7, in which *lacI* and *lacO* are inserted like pET, production of Ecb-GFP in C43(DE3) under the increased level of wild type LacI was monitored. C43(DE3) with pMW7(Ecb-GFP) could grow on 0.7 mM IPTG and the growing cells exhibit fluorescence, but C43(DE3) with pMW7::*lacI/O*(Ecb-GFP) could not (Kim *et al.*, unpublished results). It is likely that the effect of the mutant LacI produced from the chromosomal gene in C43(DE3) have been diminished by producing the wild-type LacI in excess from the plasmid.

**Figure 3:**
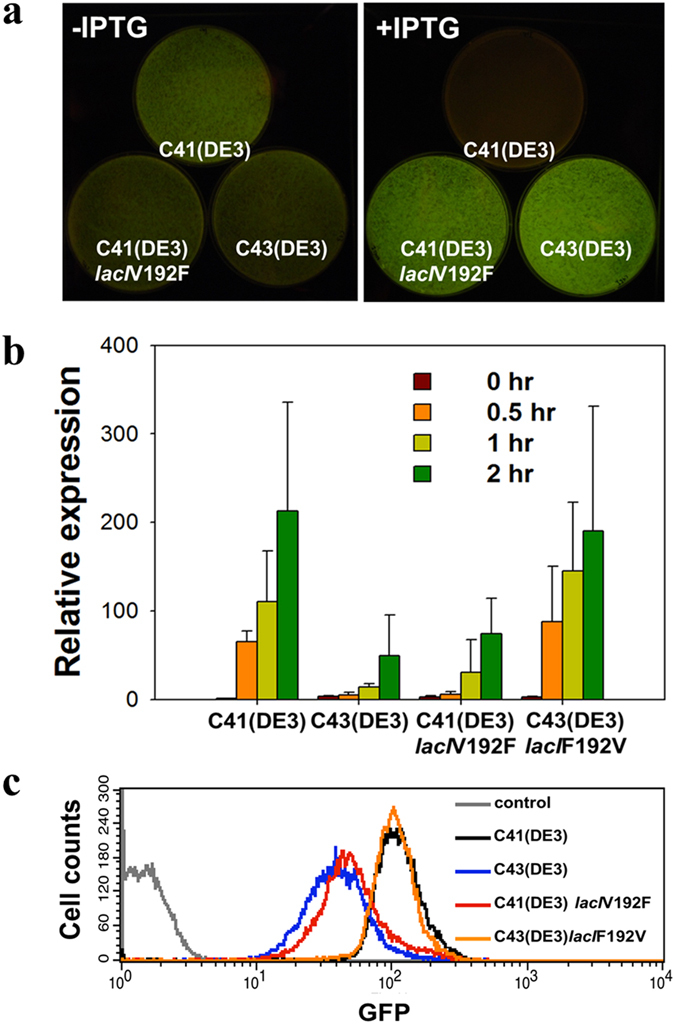
Expression capacity of *E. coli* C41(DE3), C43(DE3), C41(DE3)*lacI*V192F, and C43(DE3)*lacI*F192V to produce the *E. coli* F-ATPase subunit b. (**a**) Phenotypic comparison of Ecb expression in C41(DE3), C43(DE3), and C41(DE3)*lacI*V192F in the absence (-IPTG) or presence (+IPTG) of IPTG. C41(DE3), C43(DE3), and C41(DE3)*lacI*V192F contained pMW7(Ecb-GFP). (**b**) Relative expression levels of the *atpF* gene in C41(DE3), C43(DE3), C41(DE3)*lacI*V192F, and C43(DE3)*lacI*F192V containing pMW7(Ecb-GFP). After IPTG induction, the cultures were collected for RNA extraction. Quantification of the mRNA expression level was performed using qRT-PCR and the comparative critical threshold (2^−ΔΔCT^) method (Livak and Schmittgen, 2001). The 16S rRNA gene was used as an internal control. The transcripts at the four time points (shown on top) relative to the transcript levels in C41(DE3) at 0 hr were quantified. Error bars indicate the standard deviations of triplicate reactions. (**c**) Flow cytometric analysis of GFP fluorescence in C41(DE3) (black), C43(DE3) (blue), C41(DE3)*lacI*V192F (red) and C43(DE3)*lacI*F192V (orange) overexpressing Ecb-GFP. After 3 hours of IPTG induction, the cells were collected for detection of GFP expression. C41(DE3) without the plasmid was used as the negative control.

To verify the contribution of the *lacI* mutation to the efficient overexpression of membrane proteins in C43(DE3) and the CL strains, we introduced the mutations in *lacI* and compared the transcriptional and translational levels of Ecb-GFP. Sequence swapping of *lacI* in DE3 between C41(DE3) and C43(DE3) was performed using homologous recombination. C41(DE3) harboring *lacI*V192F and C43(DE3) with *lacI*F192V were designated C41(DE3)*lacI*V192F and C43(DE3)*lacI*F192V, respectively. C41(DE3) did not grow on L agar plates in the presence of 0.7 mM IPTG, but C41(DE3)*lacI*V192F showed fluorescence comparable with that of C43(DE3) (Fig. 3A). Cells of C41(DE3), C43(DE3), C41(DE3)*lacI*V192F, and C43(DE3)*lacI*F192V grown in L broth were collected for detailed comparison of their Ecb-GFP expression capacities after 2 hours of induction with IPTG. The quantity of the *atpF* transcript, as determined by quantitative reverse-transcription polymerase chain reaction (qRT-PCR) was approximately four-fold higher in C41(DE3) and C43(DE3)*lacI*F192V than in C43(DE3) and C41(DE3)*lacI*V192F (Fig. 3B). The Ecb-GFP expression levels as measured by flow cytometry also showed obvious differences between the two groups (Fig. 3C). Supporting our observation, LacY accumulates slower in C43(DE3) than in BL21(DE3) or C41(DE3) upon the addition of IPTG^11^.

### Structural analysis of the mutations in the LacI protein

**Figure 4:**
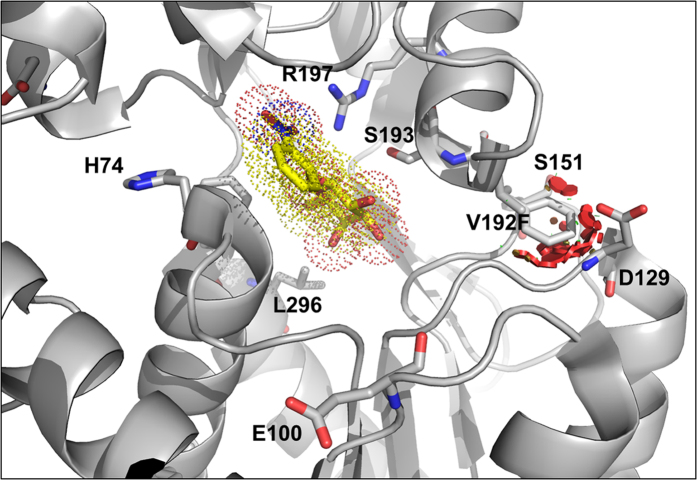
Structural analysis of the V192F mutation in the LacI protein. The model shows the steric hindrance caused by V192F, which results in changes to the sugar- or IPTG-binding pocket. Steric clashes are depicted as red disks. The model was illustrated with PyMOL.

To validate that the change from valine to phenylalanine at residue 192 of LacI is responsible for the tolerance of Ecb overexpression-induced toxicity, we introduced V192F into *lacI*-DE3 in C41(DE3) to give it the same sequence as C43(DE3). As a consequence of Ecb-GFP overexpression in C41(DE3)*lacI*V192F, the expression pattern of the recombinant protein corresponded to C43(DE3) but not to C41(DE3). Based on these results, the question remained as to how the V192F mutation in LacI counteracted toxicity. To understand the mechanism at the molecular level, the possible effects of this amino acid substitution on the structure and function of LacI were evaluated by protein homology modeling. The three-dimensional structure of LacI is available (Protein Data Bank code 1EFA); the amino acid sequence of 1EFA is identical to the LacI sequences in BL21(DE3) and C41(DE3). Therefore, V192F was introduced into the 1EFA structure and is illustrated in Fig. 4 as drawn by PyMOL^34^. As shown in the figure, V192 is located near the inducer-binding site and lies between S191 and S193, whose side chains form water-mediated hydrogen bonds with the hydroxyl group of the inducer^35^,^36^. R197, which is located in the same α-helix, is also involved in the formation of hydrogen bonds with the IPTG hydroxyl group^34^,^35^,^36^. The mutation of valine into a bulky hydrophobic phenylalanine is likely to cause a conformational change in the helix due to the introduction of steric hindrance with D129 and S151, thus resulting in weaker interactions of S191, S193 and R197 with the inducer.

Based on our analysis, some of the mutated amino acid positions in the CL strains may affect LacI binding to lactose or an allosteric effector. The R197S substitution in CL3 may reduce the binding force of hydrogen bond formation. Supporting this notion, substitution of the R197 amino acid with glycine, leucine, or lysine significantly decreased the affinity of LacI for the inducer sugars^37^. The A75T substitution in CL2 and CL3 may affect inducer stabilization as well. The nitrogen of A75 stabilizes binding to the inducer via a water-mediated hydrogen bond. The altered LacI molecules bind to the operator DNA with wild-type affinity, but do not respond to an inducer or cannot transmit the allosteric signal to the DNA binding domain; these mutant forms of LacI are classified as having the I^s^ phenotype^38^. The amino acid replacements at positions V192, R197 or A75 all produced the I^s^ phenotype.

The mutations in the LacI proteins of the C43(DE3), C41(DE3)*lacI*V192F, and CL strains may have arisen due to compromised interactions between an inducer and the repressor. Repressors that are less sensitive to the inducer are more likely to remain bound to the *lac* operator and to inhibit the transcriptional initiation of the *lac*UV5 promoter by RNAP, thereby delaying the production of mRNA encoding the membrane protein. This delay seems to be sufficient to allow the membrane protein biogenesis machinery to integrate the overexpressed proteins into the cytoplasmic membrane. The delayed induction kinetics in C43(DE3) are similar to the I^s^ phenotype^11^,^12^, and the mutation sites in these mutants belong to the mutation spectrum of the I^s^ phenotype^38^. Based on these results, we concluded that the *lacI* mutation alone can confer tolerance to membrane protein overexpression.

## Conclusions

**Figure 5:**
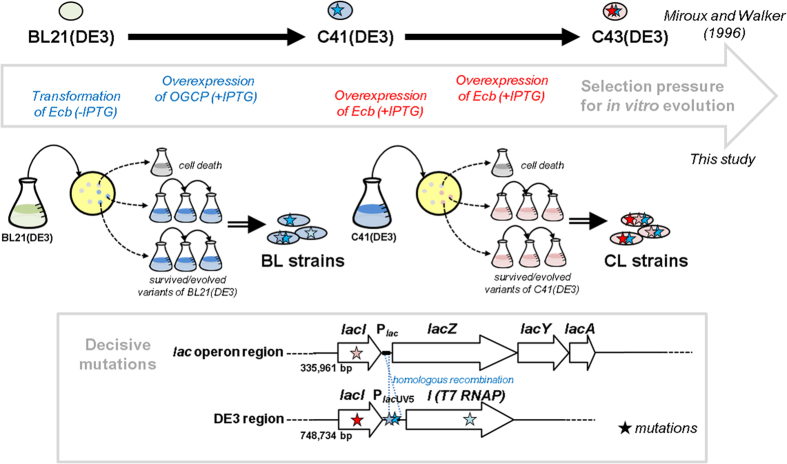
Overview of the *in vitro* evolution of *E. coli* strains overexpressing membrane proteins. A scheme to generate BL and CL strains that are capable of overcoming the toxicity from transformation with or overexpression of Ecb, respectively, are shown in the upper part of the figure. The selection of C41(DE3) from BL21(DE3), and C43(DE3) from C41(DE3), was conducted previously^12^. The mutational hotspots in the evolved strains and the probable mechanisms producing them are illustrated at the bottom. Mutational hotspots were located in the *lac*UV5 promoter of the DE3 region and in the *lacI* gene of the *lac* operon, which were identified as decisive mutations that made *E. coli* cells tolerant to the overexpression of membrane proteins (Wagner *et al.*^11^ and this study). The mutations in the *lac*UV5 promoter in the DE3 region result from homologous recombination with the similar *lac* promoter in the *lac* operon.

In summary, we identified the toxicity escape mechanisms of membrane protein-overproducing cells using a systematic approach that involved comparative genomics and evolutionary experiments. Genome-wide sequence comparisons between improved membrane protein-overproducing hosts and their ancestor provided us with a list of candidates. Parallel evolution experiments revealed the recurrence of mutations at specific genomic locations, and combined experiments involving the replacement of single amino acids in the mutated genotype allowed us to investigate the detailed mechanism at the molecular level. The evolutionary processes used to obtain superior *E. coli* hosts for the overexpression of membrane proteins are summarized in Fig. 5. Our results demonstrate that controlling the affinity of the inducer molecule is as effective as promoter engineering to reduce the toxicity caused by membrane protein overexpression with the T7 system. This ability to reduce toxicity might be pivotal for the rational design of microbial cell factories for the production of toxic foreign proteins and for membrane protein research.

## Materials and Methods

### Strains, plasmids, and culture conditions

*E. coli* strains were cultured at 37 °C in L broth. The medium was supplemented with 100 μg/ml of Amp if the cells harbored pKD46, pREDI, or derivatives of pMW7. To construct pMW7(Ecb-GFP), we used the GFP-encoding gene sequence described in Waldo *et al.*^39^ When designing the primers for PCR amplification, *Nde*I and *Bam*HI sites at the 5’ end of the GFP gene and a *Hin*dIII site at the 3′ end were added for efficient cloning. The amplified sequence was cloned into pMW7 digested with *Nde*I/*Hin*dIII to yield pMW7(GFP). Ecb was amplified with a T7-promoter primer and Ecb(NSBamHI)-R to remove the stop codon. The amplified product was cloned into the *Nde*I/*Bam*HI site of pMW7(GFP) to yield pMW7(Ecb-GFP). All of the constructs were confirmed by sequencing. The bacterial strains and plasmids used in this study are listed in Supplementary Table 4, and the primers are listed in Supplementary Table 5.

### Comparative genome analysis of *E. coli* C41(DE3), C43(DE3), and BL21(DE3)

Roche/454 pyrosequencing was conducted to identify the genomic variations in C41(DE3) and C43(DE3) with respect to BL21(DE3). Chromosomal DNA was prepared using the Wizard® Genomic DNA Purification Kit (Promega, U.S.A.) according to the manufacturer’s instructions. Shotgun reads with approximately 12.2-fold and 9.5-fold genome coverage were generated using GS FLX for C41(DE3) and C43(DE3), respectively (NICEM, Korea). The reads were then mapped onto the reference genome sequence of BL21(DE3) (GenBank ID CP001509). The gsAssembler (version 2.0.00) program was used to detect SNPs. To detect large-scale insertions, deletions or conversions, the reads were *de novo* assembled using gsAssembler. The consensus sequence of each contig was converted into individual pseudoreads^40^. The PHRED/PHRAP/CONSED package was used to call the bases of the pseudoreads, to map the pseudoreads to the BL21(DE3) reference sequence, and to detect large-scale genomic variation^41^,^42^.

Additionally, the sequence information for C43(DE3), which reached 890-fold genome coverage, was obtained using an Illumina Solexa Genome Analyzer II. This high-depth coverage enabled more-detailed detection of variations in the C43(DE3) genome by overcoming the homopolymer problem that is encountered in pyrosequencing methods^43^. Reads from Solexa sequencing were mapped to the BL21(DE3) standard sequence, and a genomic variation search was conducted. All of these steps were conducted using CLC genomics workbench (CLC bio, Inc., Denmark). All of the detected candidate regions containing variations were amplified by PCR and confirmed by Sanger sequencing. Furthermore, we selected 10 random colonies of C41(DE3) and C43(DE3) to distinguish genuine mutations from individual clonal variations. PCR amplification and targeted sequencing were then performed for the regions corresponding to the mutated regions. The primers used for PCR and Sanger sequencing are listed in Supplementary Table 6. The raw sequence reads and the genome information of *E. coli* C41(DE3) have been deposited at Short Reads Archive and GenBank under the accession numbers SRR2050391 and LFIN00000000. Those of and C43(DE3) are SRR2052575, SRR2052522 and CP011938.

### λ Red-mediated homologous recombination

All of the mutation-introducing methods in this study are based on λ Red-mediated homologous recombination. Here, we describe the construction process of C41(DE3)*lacI*V192F as a representative case. Individual mutations were introduced into C41(DE3) and C43(DE3) through the two steps of λ Red-mediated homologous recombination. To construct the C41(DE3)*lacI*V192F, the *lacI* region of C41(DE3) was replaced with that of C43(DE3). First, a PCR fragment targeting the *lacI* gene disruption was generated. The target fragment was designed to contain a 40-bp region homologous to *lacI* at both ends with a chloramphenicol resistance gene flanked by I-*Sce*I recognition sites. PCR was conducted with the lacIKO-F and lacIKO-R primers using pKD3-IsceI as the template. pKD3/I-*Sce*I is a pKD3-backbone plasmid in which I-*Sce*I recognition sites are inserted on both sides of the chloramphenicol resistance gene. The method described in Datsenko *et al.* was used for transforming the PCR-targeted fragment into C41(DE3) harboring the λ Red helper plasmid pKD46 and for selecting mutants^44^. After primary selection, the mutants were maintained at 37 °C without Amp and then tested for Amp sensitivity to detect the loss of the helper plasmid pKD46. Next, the pREDI plasmid was introduced into *lacI*-disrupted C41(DE3) to express the homing endonuclease I-*Sce*I^45^. Using recombination mediated by I-*Sce*I cleavage and subsequent double-stranded break repair, we induced the replacement of the *lacI* allele in the DE3 region of C43(DE3) by transformation with the PCR-targeted fragment. Two *lacI* genes exist in C43(DE3). PCR was conducted with the DE3 region-specific primers T7lacI-F and lacI-R. The resulting product was then used as a template for target fragment PCR with the primers T7lacIS-F and T7lacIS-R. Transformation of the PCR-targeted fragment and rhamnose induction of the I-*Sce*I endonuclease was conducted using the method described in Yu *et al.*^45^ Recombination was confirmed by PCR, which showed a size difference in the T7lacI-F and lacI-R products.

### Experimental evolution studies

#### BL strains

BL21(DE3) was transformed with pMW7(Ecb)^12^, or pMW7(Ecb-GFP), which express Ecb or GFP-fused Ecb, respectively, under the control of the T7 promoter. After transformation, the cells were plated on L agar medium supplemented with Amp. Two sub-populations composed of either large or small colonies formed. The small colonies could not grow on a plate with Amp but without IPTG during subsequent culturing, whereas the large colonies survived. The cells from the large colonies were termed BL, for large colonies obtained from BL21(DE3). BL strains were cured of pMW7(Ecb) or pMW7(Ecb-GFP) by growth on an L agar plate in the absence of Amp. Therefore, the mutation affecting survival after transformation of pMW7(Ecb) was located in the chromosome and not in the plasmid itself.

#### BR strains

Two homologous merodiploid regions are from *lacI* to the partial *lacZ* gene in BL21(DE3) and C41(DE3), one in the DE3-lysogenized region and the other at the original *lac* locus; the two regions have a four-nucleotide difference. We constructed two knockout strains, BL21(DE3)Δ*lacI-*P_*lac*_ and C41(DE3)Δ*lacI-*P_*lac*_, in which the upstream 1,723 bp of the *lac* operon were deleted by λ Red-mediated homologous recombination. These strains were transformed with pMW7(Ecb-GFP). After transformation, the cells from the cultures were plated on L agar with Amp and 0.7 mM IPTG. The surviving transformants of BL21(DE3)Δ*lacI-*P_*lac*_ were named BR, for BL21(DE3) recombinants. The chromosomal nature of the mutations in the BR strains was confirmed by loss and re-introduction of the plasmid.

#### CL strains

pMW7(Ecb-GFP) was transformed into C41(DE3), and the cells from the culture were cultivated on L agar with Amp and 0.1 mM IPTG. Twenty-three colonies arose as a result. Among the colonies, three showed similar growth and fluorescence intensity to that of C43(DE3) during subsequent cultivation. We named these mutants CL, for large colonies obtained from C41(DE3). The CL strains were cured of pMW7(Ecb-GFP) by repeated cultivation in the absence of Amp, confirming that the mutation affecting the expression of Ecb-GFP was present only in the chromosome and not in the pMW7(Ecb-GFP) plasmid.

### Measurement of gene expression levels

To analyze cells expressing Ecb-GFP, the expression levels of the *gfp* gene and the recombinant gene were quantitatively measured. Triplicate cultures of C41(DE3), C43(DE3), C41(DE3)*lacI*V192F, and C43(DE3)*lacI*F192V harboring pMW7(Ecb-GFP) were grown in 50 ml of L broth with Amp. When the culture optical densities at 600 nm reached approximately 0.4, 3 ml of the culture was centrifuged, and the pellet was stored immediately in 500 μl of RNAlater (Ambion, U.S.A.) to yield the Time 0 sample. For the remaining cultures, IPTG was added to each cell culture to a final concentration of 0.7 mM. Each culture was collected in 500 μl of RNAlater by the same method after 0.5, 1, or 2 hours. The optical density at 600 nm was measured using a UV/Vis spectrophotometer (Optizen, Korea) at 1-hour intervals.

Two hours after IPTG addition, the GFP intensity of each cell was measured using flow cytometry on a FACSCalibur instrument (BD Biosciences, U.S.A.). The cultures were diluted in PBS to a final concentration of 10^6^ cells/ml immediately after harvesting. A low flow rate was used throughout data collection, with an average of 250 events/s. The cellular accumulation levels of the GFP fusion proteins were measured by GFP fluorescence intensity. Data acquisition was performed using CellQuest software (BD Biosciences), and the data were analyzed using FloJo software (Tree Star).

To assess the gene expression of Ecb, all of the RNAlater-treated samples were stored at 4 °C until RNA extraction using the RNeasy kit (Qiagen, U.S.A.). The remaining chromosomal DNA was eliminated using the Turbo DNA-free kit (Ambion, U.S.A.). No amplification was observed after 40 cycles of PCR when using the RNA samples as templates without the reverse transcription step, confirming complete removal of DNA. cDNA was obtained for RT-PCR by using the M-MLV cDNA Synthesis Kit (Enzynomics, Korea). Real-time PCR was performed on a CFX Connect™ Real-Time PCR Detection System (Bio-Rad, U.S.A.) with iQ™ SYBR® Green Supermix (Bio-Rad). Each cDNA sample was amplified with specific primers, as follows: qBL21_16S1-F/R were used for the internal 16S rRNA control and qBL21_16S1-F/R were used for the *atpF* gene. All reactions were quantified in triplicate. The PCR conditions included an initial denaturation step at 95 °C for 5 min, followed by 40 cycles of amplification at 95 °C for 30 s, 55 °C for 30 s, and 72 °C for 1 min. Finally, an additional step to establish the melting curve was performed, in which the temperature was decreased from 95 °C to 65 °C (0.05 °C s-1). Threshold cycle (Ct) values for each measurement were determined. The relative quantification of gene expression was calculated using the comparative critical threshold 2^−ΔΔC^_T_ method, in which the amount of the RNA of interest is normalized to an internal reference RNA^46^. The 16S rRNA gene was used as an internal control in this study. The following equations were used:

ΔC_T_ = Ct of the *atpF* gene (gene of interest)–Ct of the 16S rRNA gene (internal control).

ΔΔC_T_ = ΔC_T_ of the samples after IPTG induction – ΔC_T_ of the Time 0 sample.

Relative expression level = 2^−ΔΔCt^.

## Additional Information

**How to cite this article**: Kwon, S.-K. *et al.* Comparative genomics and experimental evolution of *Escherichia coli* BL21(DE3) strains reveal the landscape of toxicity escape from membrane protein overproduction. *Sci. Rep.* **5**, 16076; doi: 10.1038/srep16076 (2015).

## Supplementary Material

Supplementary Information

## Figures and Tables

**Table 1:** Annotated functions of the genetic changes in *E. coli* C41(DE3) and C43(DE3).

Gene	Function	Mutation	Gene position	Region	Effect
*Present only in* C41(DE3)					
*proY*	Predicted cryptic proline transporter	T → A	438	Coding	Synonymous
*melB*	Melibiose:sodium symporter	C → T	653	Coding	Ala → Val
*ycgO*	Potassium/proton antiporter	C → A	1103	Coding	Gly → Val
*yhhA*	Hypothetical protein	C → T	290	Coding	Pro → Leu
*Present only in* C43(DE3)					
*dcuS*	Sensor of fumarate two-component system; frame-shifted in BL21(DE3)	GCGCC deletion	866–870	Coding	Frameshift
*fur*	Ferric uptake regulator	GTC insertion	392	Coding	Val insertion
*lacI*	*lac* repressor	G → T	574	Coding	Val → Phe
*lon*	DNA-binding ATP-dependent protease La	IS*4* excision	−156 from *lon*	Intergenic	Activation of Lon protease
*yibJ*	Predicted Rhs-family protein	C → A	90	Coding	Synonymous
*yjcO*	Hypothetical protein	A → G	665	Coding	Glu → Gly
*cydA*	Cytochrome d terminal oxidase, subunit I	IS*1* insertion	−262 from *cydA*	Promoter	N.D.
*ccmF* ~ ompC*	Listed in Supplementary Table 1	IS*1* deletion	21-kb large deletion	N.D.	
*yjiV* ~ yjjN*	Listed in Supplementary Table 2	IS*1* deletion	37-kb large deletion	N.D.	
*Present in both* C41(DE3) *and* C43(DE3)					
*l*	T7 DNA-directed RNA polymerase	AA −> GT, G −> A	P_*lac*UV5_ −10 P_*lac*UV5_ CAP binding site	Promoter	
*rbsD*	Predicted cytoplasmic sugar-binding protein; disrupted by insertion of IS in BL21(DE3)	IS*3* excision	217–1586	Coding	N.D.
*yehU*	Predicted sensory kinase in two-component system	T −> G	679	Coding	Phe → Val

N.D. not determined.

^*^Detailed information on the genes in the region of the large-scale genomic deletion is listed in the Supplementary tables.

**Table 2:** Sequence differences in the promoter region and coding sequence of T7 RNAP.

Strain/Group	Promoter sequence of T7 RNAP		Coding sequence of T7 RNAP	No. of colonies
	CAP binding site	−10 to +1 region		
BL21(DE3)	P_*lac*UV5_	P_*lac*UV5_	—	—
C41(DE3) C43(DE3)	P_*lac*UV5_	P_*lac*_	—	—
BL1^†^	P_*lac*UV5_	P_*lac*_	N.D.	3
BL2^†^	P_*lac*_	P_*lac*_	N.D.	9
BL3^†^	P_*lac*UV5_	P_*lac*UV5_	changed	3

BL, large colonies obtained from BL21(DE3).

^†^Evolved mutants obtained in this study.

N.D., not determined.

**Table 3:** Sequence differences in the *lacI* gene.

Strain/Group	Genomic location	
	DE3 phage-inserted	*lac* operon
C43(DE3)	G574T (V192F)	No mutation
CL1†	C583A (R197S)	No mutation
CL2†	No mutation	G223A (A75T)
CL3†	G673A (G225S)	G223A (A75T)

CL, large colonies obtained from C41(DE3).

^†^Evolved mutants obtained in this study.
