# Supplementary material for: Comparative genomics and experimental evolution of Escherichia coli BL21(DE3) strains reveal the landscape of toxicity escape from membrane protein overproduction
Source: Sci Rep. 2015 Nov 4;5:16076. doi: 10.1038/srep16076 (PMC4632034; doi:10.1038/srep16076)

Comparative genomics and experimental evolution of *Escherichia coli* BL21(DE3) strains reveal the landscape of toxicity escape from membrane protein overproduction

Soon-Kyeong Kwon[1,3†], Seong Keun Kim[2,3†], Dae-Hee Lee[2,3], and Jihyun F. Kim[1*]

[1]Department of Systems Biology and Division of Life Sciences, Yonsei University, 50 Yonsei-ro, Seodaemun-gu, Seoul 120-749, Republic of Korea; [2]Synthetic Biology and Bioengineering Research Center, Korea Research Institute of Bioscience and Biotechnology (KRIBB), 125 Gwahak-ro, Yuseong-gu, Daejeon 305-806, Republic of Korea; [3]Biosystems and Bioengineering Program, University of Science and Technology (UST), 217 Gajung-ro, Yuseong-gu, Daejeon 305-350, Republic of Korea

[†]These authors contributed equally to this work.

[*] Correspondence to: Jihyun F. Kim, Department of Systems Biology, College of Life Science and Biotechnology, Yonsei University, 50 Yonsei-ro, Seodaemun-gu, Seoul 120-749, Korea. Tel: +82 2 2123 5561; Fax: +82 2 312 5657; E-mail: jfk1@yonsei.ac.kr.

Supplementary Table 1. Bacterial strains and plasmids used in this study.

Strain or plasmid	Derivation, description, and/or phenotype	Source or reference
**Bacterial strains**		
Escherichia coli		
BL21(DE3)	*E. coli* B F^- *dcm ompT hsdS*(r_B^- m_B^-) *gal lon* λ(DE3 [*lacI lac*UV5-T7 gene *1 ind1 sam7 nin5*])	(Studier and Moffatt, 1986)
C41(DE3)	Derived from BL21(DE3) by selecting for resistance to OGCP overexpression	(Miroux and Walker, 1996)
C43(DE3)	Derived from C41(DE3) by selecting for resistance to Ecb overexpression	(Miroux and Walker, 1996)
BL strains	BL21(DE3) derivatives overcoming the toxicity from Ecb transformation	This study
BL21(DE3)Δ*lacI*-P_{lac}	Variant of BL21(DE3) where *lacI* to part of *lacZ* is deleted	This study
C41(DE3)Δ*lacI*-P_{lac}	Variant of C41(DE3) where *lacI* to part of *lacZ* is deleted	This study
BR strains	BL21(DE3)Δ*lacI*-P_{lac} derivatives overcoming the toxicity from Ecb overexpression	This study
CL strains	C41(DE3) derivatives overcoming the toxicity from Ecb overexpression	This study
C41(DE3) *lacI*V192F	V192F mutation in the *lacI* gene at the DE3 region	This study
C43(DE3) *lacI* F192V	F192V mutation in the *lacI* gene at the DE3 region	This study
**Plasmids**		
pKD46	Plasmid expressing λ-Red, P_{araB}, Amp^R	(Datsenko and Wanner, 2000)
pREDI	P_{araB} λ-Red, P_{rhaB} I-*Sce*I endonuclease expressing plasmid, Amp^R	(Yu et al., 2008)
pKD3	Template plasmid for gene disruption	(Datsenko and

	with Cm resistance gene flanked by FRT sites, Cm^R, Amp^R	Wanner, 2000)
pKD3/I-*Sce*I	pKD3, I-*Sce*I endonuclease recognition site introduced at both side of the Cm resistance gene, Cm^R, Amp^R	This study
pMW7(OGCP)	T7 promoter, bovine oxoglutarate-malate transport protein (OGCP) cloned pMW7, Amp^R	(Miroux and Walker, 1996)
pMW7(Ecb)	T7 promoter, *E. coli* F-ATPase subunit b (Ecb) cloned in pMW7, Amp^R	(Miroux and Walker, 1996)
pMW7(GFP)	pMW7 with GFP inserted in the *Bam*HI/*Hin*dIII site	This study
pMW7(Ecb-GFP)	pMW7 with Ecb fused to GFP at the C-terminus; inserted in the *Nde*I/*Hin*dIII site	This study

Supplementary Table 2. Primers used for cloning or constructing mutants.

Name	Sequence (5' to 3')
T7 promoter primer	TAATACGACTCACTATAGGG
Ecb(NSBamHI)-R	GGGGATCCCAGTTCAGCGACAAGTTTT
lacIKO-F	TCGCATTGGGTCACCAGCAAATCGCGCTGTTAGCGG GCCCGGTCCATATGAATATCCTCC
lacIKO-R	CAACCAGCATCGCAGTGGGAACGATGCCCTCATTCA GCATGGAATACGGTTAGCCATTTG
T7lacI-F	TTGCTCCGGGCTATGAAATA
lacI-R	GTTTTCCCAGTCACGACGTT
T7lacIS-F	CCGGCGTTATTTCTTGATGT
T7lacIS-R	CGGTATCGTCGTATCCCACT
qBL21_16S1-F	CGTGTATGAAGAAGGCCTTCG
qBL21_16S1-R	CTGAGCGTCAGTCTTCGTCC
qECB1-F	CCTGTTCGTTCTGTTCTGCATG
qECB1-R	ACTTGCTTACGCAGCTCTTCAC

Supplementary Table 3. Primers used to confirm the mutations in C41(DE3) or C43(DE3).

Name	Sequence (5' to 3')
dcu_F/R	CACAGTGCAGCCAGCCG/<br>TGATTGGCACCTGCATTCTGG
fur_F/R	GGCTTTCTCGTTCAGGCTGGC/<br>ATGACTGATAACAATACCGCCC
lacI_operon_F/R	ACTGTCCTGGCCGTAACCGA/<br>CAGGGTGGTGAATGTGAAACC
lon_F/R	CCATTCCCATACAATTAGTTAACC/<br>CGGATAGATTTTTCCCGCCCG
mel_F/R	GATCGGTACGTTGGCAAACTCT/<br>GGAAGTAGAGAATCAGCGTTACCAT
rbs_F/R	TGATATTTCATCGGTGATCTCCC/<br>CGAATTTCAATGGTATTTCCCTG
lac_DE3_F/R	GAAAGGTAGGCGGATCCAGATC/<br>CCTCTTCCAGTTAGTAAATCCGGA
ycg_F/R	GATTCAGCTTCCTCTTCAGCCA/<br>GTTTCAATCTGCGTGAGCGC
yeh_F/R	CGGATCACCGCTTTAATGGTG/<br>GTAATGGGCGGCTTACTCGG
yhh_F/R	ATGAAACGACTTCTGATTCTTACGG/<br>ATACCGTCAATAAACCCCAGCG
yib_F/R	TAAGCTGAAGCGTTCATAGCGTG/<br>TTTATATCAACCAAACCTTGCGG
yjc_F/R	GTAGGTCGGATAAGGCGTTTACG/<br>TTTAGCTCCGGCGATTTGAG
ync+cyd_F/R	CAAACCGAAGCCACATATGCG/<br>GCAAACTGTAAGCGCGACAG
ccm+omp_F/R	GTCCAGTGATTCGAGACGAAAC/<br>GCCGCATCCGGCATTCAG
yji+yjj_F/R	CGAATTTGCCGACCCGACTA/<br>CGCCGCGTAAGCTCGGG

Supplementary Table 4. List of genes included in a large deletion across *ccmF* ~ *ompC* in C43(DE3) and their annotated functions.

Gene	Function
ccmF	heme lyase, CcmF subunit
ccmE	periplasmic heme chaperone
ccmD	cytochrome c biogenesis protein
ccmC	heme exporter subunit
ccmB	heme exporter subunit
ccmA	heme exporter subunit
napC	nitrate reductase, cytochrome c-type, periplasmic
napB	nitrate reductase, small, cytochrome C550 subunit, periplasmic
napH	quinol dehydrogenase membrane component
napG	quinol dehydrogenase periplasmic component
napA	nitrate reductase, periplasmic, large subunit
napD	assembly protein for periplasmic nitrate reductase
napF	ferredoxin-type protein, predicted role in electron transfer to periplasmic nitrate reductase
eco	ecotin precursor
mqo	malate:quinone oxidoreductase
yojI	fused predicted multidrug transport subunits of ABC superfamily: membrane component/ATP-binding component
alkB	oxidative demethylase of N1-methyladenine or N3-methylcytosine DNA lesions
ada	fused DNA-binding transcriptional dual regulator/O6-methylguanine-DNA methyltransferase
yojL	predicted thiamine biosynthesis lipoprotein
ompC	outer membrane porin protein C

Supplementary Table 5. List of genes included in a large deletion across *yjiV* ~ *yjjN* in C43(DE3) and their annotated functions.

Gene	Function
yjiV	hypothetical protein
mcrC	5-methylcytosine-specific restriction enzyme McrBC, subunit McrC
mcrB	5-methylcytosine-specific restriction enzyme McrBC, subunit McrB
yjiW	hypothetical protein
hsdS	interrupted by IS*1*; specificity determinant for hsdM and hsdR
hsdM	DNA methylase M
hsdR	endonuclease R
mrr	methylated adenine and cytosine restriction protein
yjiA	predicted GTPase
yjiX	hypothetical protein
yjiY	predicted inner membrane protein
hpaC	4-hydroxyphenylacetate 3-monooxygenase, reductase component
hpaB	4-hydroxyphenylacetate 3-monooxygenase
hpaA	hypothetical protein
hpaX	hypothetical 4-hydroxyphenylacetate permease
hpaI	2,4-dihydroxyhept-2-ene-1,7-dioic acid aldolase
hpaH	2-oxo-hept-3-ene-1,7-dioate hydratase
hpaF	5-carboxymethyl-2-hydroxymuconate delta-isomerase
hpaD	Homoprotocatechuate dyoxygenase
hpaE	5-carboxy-2-hydroxymuconate semialdehyde dehydrogenase
hpaG	4-hydroxyphenylacetate degradation bifunctional isomerase/decarboxylase
hpaR	Homoprotocatechuate degradative operon repressor
tsr	methyl-accepting chemotaxis protein I, serine sensor receptor
yjiZ	predicted transporter
yjjM	frameshift; putative transcriptional regulator
yjjN	predicted oxidoreductase, Zn-dependent and NAD(P)-binding

Supplementary Table 6. List of all nonsynonimous mutations in C43(DE3) and strains obtained from parallel evolution of BL21(DE3) and C41(DE3).

Strains	Changes	Position
BL3-1	CTT -> TTT (L791F)	T7 RNAP coding gene *l*
BL3-2	TCA -> TA	T7 RNAP coding gene *l*
BL3-3	CAA -> TAA	T7 RNAP coding gene *l*
BR-1	CGC -> TGC (R829C)	T7 RNAP coding gene *l*
BR-2	AGC -> AAC (S785N)	T7 RNAP coding gene *l*
BR-3	GGT -> GAT (G456D)	T7 RNAP coding gene *l*
BR-4	GGT -> GAT (G456D)	T7 RNAP coding gene *l*
C43(DE3)	GTC-> TTC (V192F)	*lacI* in DE3 phage-inserted
CL1	GGT-> AGT (G225S)	*lacI* in DE3 phage-inserted
CL2	GCG-> ACG (A75T)	*lacI* in *lac* operon
CL3	GCG-> ACG (A75T)	*lacI* in *lac* operon
CL3	CGT-> AGT (R197S)	*lacI* in DE3 phage-inserted

Supplementary Figure 1. Transformants of BL21(DE3) Δ*lacI*-P$_{lac}$ and C41(DE3) Δ*lacI*-P$_{lac}$ carrying the pMW7 vector expressing GFP-fused Ecb.

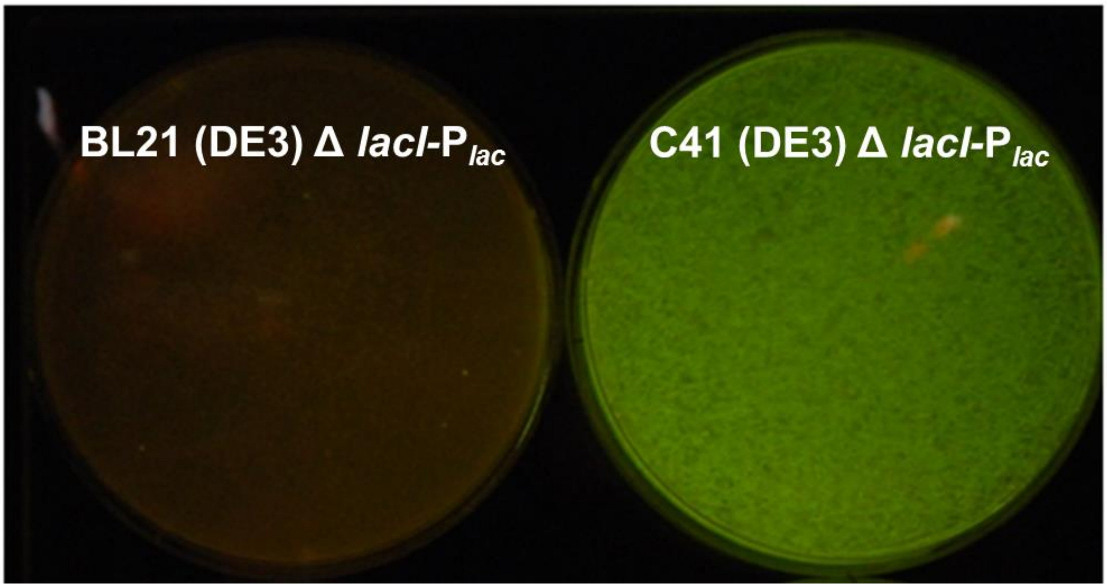

Supplement: Supplementary Information [file srep16076-s1.pdf]
